# JAK Inhibition in a Patient with X-Linked Reticulate Pigmentary Disorder

**DOI:** 10.1007/s10875-020-00867-7

**Published:** 2020-09-28

**Authors:** Corinne Légeret, Benedikt J. Meyer, Annette Rovina, Nikolaus Deigendesch, Christoph T. Berger, Thomas Daikeler, Ingmar Heijnen, Ezra Burstein, Henrik Köhler, Mike Recher

**Affiliations:** 1 Department of Gastroenterology, Children’s Hospital Aarau, Aarau, Switzerland; 2 Immunodeficiency Clinic and Immunodeficiency Laboratory, Medical Outpatient Unit and Department of Biomedicine, University Hospital Basel, Basel, Switzerland; 3 Department of Pathology, University Hospital Basel, Basel, Switzerland; 4 Translational Immunology, Department of Biomedicine, University Hospital Basel, Basel, Switzerland; 5 Rheumatology Clinic, University Hospital Basel, Basel, Switzerland; 6 Division Medical Immunology, Laboratory Medicine, University Hospital Basel, Basel, Switzerland; 7 Departments of Internal Medicine and Molecular Biology, UT Southwestern Medical Center, Dallas, TX USA

To the editor,

X-linked reticulate pigmentary disorder (XLPDR) is a very rare inherited disease with prominent skin hyperpigmentation and multiorgan involvement dominated by autoinflammatory manifestations in the eyes, the urinary tract, and recurrent infections, particularly in the respiratory tract. In 2016, the causative genetic mutation for XLPDR was located to the *POLA1* gene, which encodes the catalytic subunit of DNA polymerase A1 [1]. An intronic *POLA1* mutation (c.1375-354A>G), causing altered *POLA1* gene splicing, leads to reduced transcript and protein levels. It has been demonstrated that POLA1 deficiency is directly linked to the activation of type I interferons (IFNs) and upregulation of interferon-stimulated genes (ISG), classifying XLPDR as an interferonopathy [1]. In affected males, the disease typically manifests in the first months of life with failure to thrive, recurrent pneumonias, persistent diarrhea in infancy, and pathognomonic diffuse hyperpigmentation and characteristic facies [2].

The patient described here was born in 2008 into a Caucasian, non-consanguineous family via spontaneous vaginal delivery at term. The index patient has two older, healthy brothers and healthy parents. His mother lacked pigmentary changes along Blaschko’s lines. At the age of four weeks, he was admitted to the hospital due to low weight for age (Fig. S1). During the admission, he developed mild bloody diarrhea. All laboratory tests performed at that time (blood count, electrolytes, liver parameters, clotting time, thyroid function, viral serologies, screening for inborn metabolic disorders, stool screenings for malabsorption, and infections) were within reference ranges. The hypothesis of a cow’s milk protein allergy was made, the formula was swapped to an amino acid based one, the symptoms resolved, and the patient started to gain weight. Since the age of six months, his weight follows percentiles 3–15 (Supplementary Fig. 1). On clinical examination during the hospitalization, a mild muscular hypotonia was noticed and further tests (MRI of the head, abdominal ultrasound, EEG, and referral to the ophthalmologist) were arranged—all with normal results. Neuropediatric follow-up was arranged due to the mild muscular hypotonia but was stopped at the age of 2 years because a normal development was objectified. Over the next years, the patient suffered from recurrent otitis media, sinusitis, and several pneumonias. Despite the performance of an adenoidectomy, tympanocentesis, and dacryocystorhinostomy at the age of four, a purulent obstructive rhinosinusitis persisted. Primary ciliary dyskinesia was excluded by biopsy, and no disease-associated mutations were detected in the cystic fibrosis (CFTR) gene. Skin prick testing for allergies was negative. After re-adenoidectomy and infundibulotomy, a local symptomatic treatment was continued until today.

In addition to the respiratory symptoms, the patient suffered from recurrent abdominal pain and intermittently diarrhea since the age of 18 months. Several abdominal ultrasounds and stool laboratory tests (including calprotectin) did not reveal any abnormal findings at first. Fecal calprotectin was elevated for the first time (595 μg/g, reference < 100 μg/g) at the age of 4 years. A panendoscopy at that time demonstrated only mild rectal Paneth cell metaplasia. After an initial good response to mesalazine, abdominal pain re-occurred at the age of 7 years. Gastrointestinal biopsies now demonstrated increased intraepithelial apoptosis and eosinophilic inflammation, most pronounced in the ascending colon, requiring systemic steroids (Fig. 1d). The dermal appearance (upswept hairline and hyperpigmentation in the periorbital region, around the shoulders, and on the trunk, Fig. 1a), combined with recurrent airway infections plus inflammatory bowel disease suggested the possibility of XLPDR which was first considered at the age of 6 years. The previously described disease-causing hemizygous c.1375-354A>G *POLA1* mutation was confirmed in February 2019 by targeted sequencing.

**Fig. 1:**
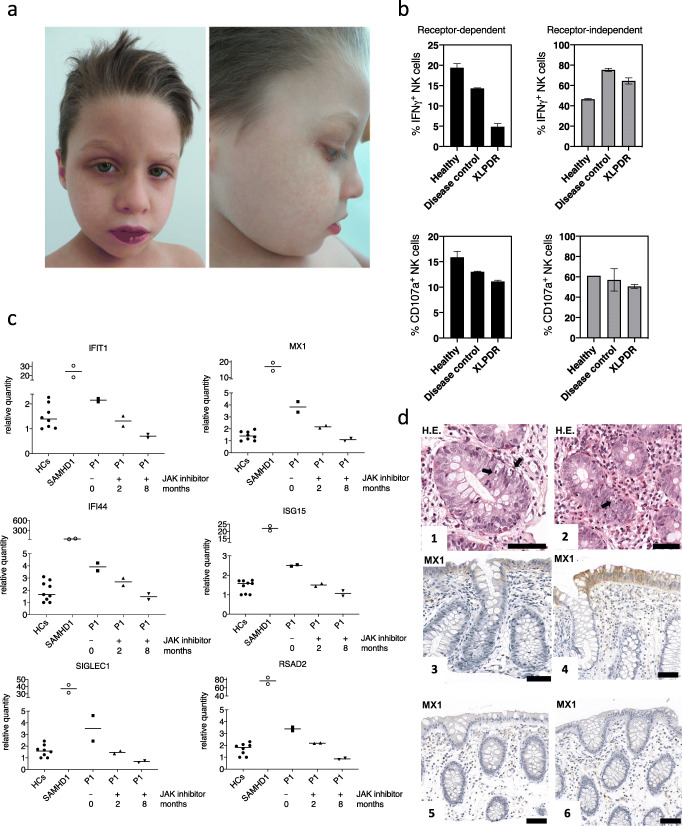
(a) Image displaying hyperpigmentation, upswept hair, and flared eyebrows in the patient with XLPDR. (b) PBMC of the patient with XLPDR, a healthy control or a patient with recurrent infections of not yet determined cause (Disease control) were stimulated in vitro with PMA/ionomycin or incubated with K562 tumor cells that lack MHCI. Six hours later, intracellular IFN-γ formation was assessed by flow cytometry. Alternatively, cell surface expression of CD107a was assessed by flow cytometry. The mean and standard deviation of two replicate measurements is depicted. (c) Interferon-stimulated genes (ISG) were assessed by real-time PCR from whole-blood cDNA of the XLPDR patient (P1) before therapy (squares), or under tofacitinib therapy for 2 months (triangle up) or 8 months (triangle down) in comparison to 8 different healthy control individuals (HCs, filled circles) and to a patient with a SAMHD1-related interferonopathy (SAMHD1, open circles). Gene expression of *IFIT1*, *MX1*, *IFI44*, *ISG15*, *SIGLEC1*, and *RSAD2* was assessed. Pooled data from repetitive experiments are shown. Data normalized to actin using the delta-delta-CT method. Values are shown as fold change to a specific healthy control sample, which was used as the normalization control in every experiment. Symbols representing the XLPDR patient depict ISG measured in two independent experiments from the same cDNA. Symbols representing healthy control individuals depict ISG measured in 8 different healthy control cDNA. (d) Pictures 1+2: hematoxylin-eosin (H.E.) staining of ascending colon biopsies of the patient with XLPDR. Arrows mark intraepithelial apoptosis. Pictures 3–6: immuno-histological staining for MX1 in ascending colon biopsies of the patients with XLPDR [3], a patient with non-POLA1 associated inflammatory bowel disease [4], and two control individuals without colon inflammation [5+6]. The black line (inlet) represents a distance of 50 μm

Following the molecular confirmation of XLPDR, a comprehensive immunologic evaluation was performed: serum immunoglobulins including IgG subclasses were within reference values for age (Table 1). NK cell count was slightly low in absolute numbers, while total B and T cell numbers were normal. B and T cell subpopulations were normal for age, and the oxidative burst test (DHR123) revealed normal neutrophil burst activity (Table 1). It has been recently demonstrated that NK cells are dysfunctional in patients with XLPDR [3]. Therefore, the NK cell phenotype and function in the XLPDR patient were evaluated compared to a simultaneously recruited healthy (adult) control and an adult patient with recurrent infections with possible, but not yet further evaluated primary immunodeficiency (Disease control). The relative proportion of immature CD56^hi^ NK cells was 2-fold elevated compared to the two controls (Fig. S2), in keeping what has been described in other XLPDR patients [3]. NK function was assessed flow cytometrically by measuring intracellular IFN-γ and CD107a in response to receptor-dependent (K562 cells) and receptor-independent (PMA/ionomycin) activation (Fig. 1b). The XLPDR patient showed a specifically reduced NK cell response to receptor-dependent activation (Fig. 1b).

**Table 1:**
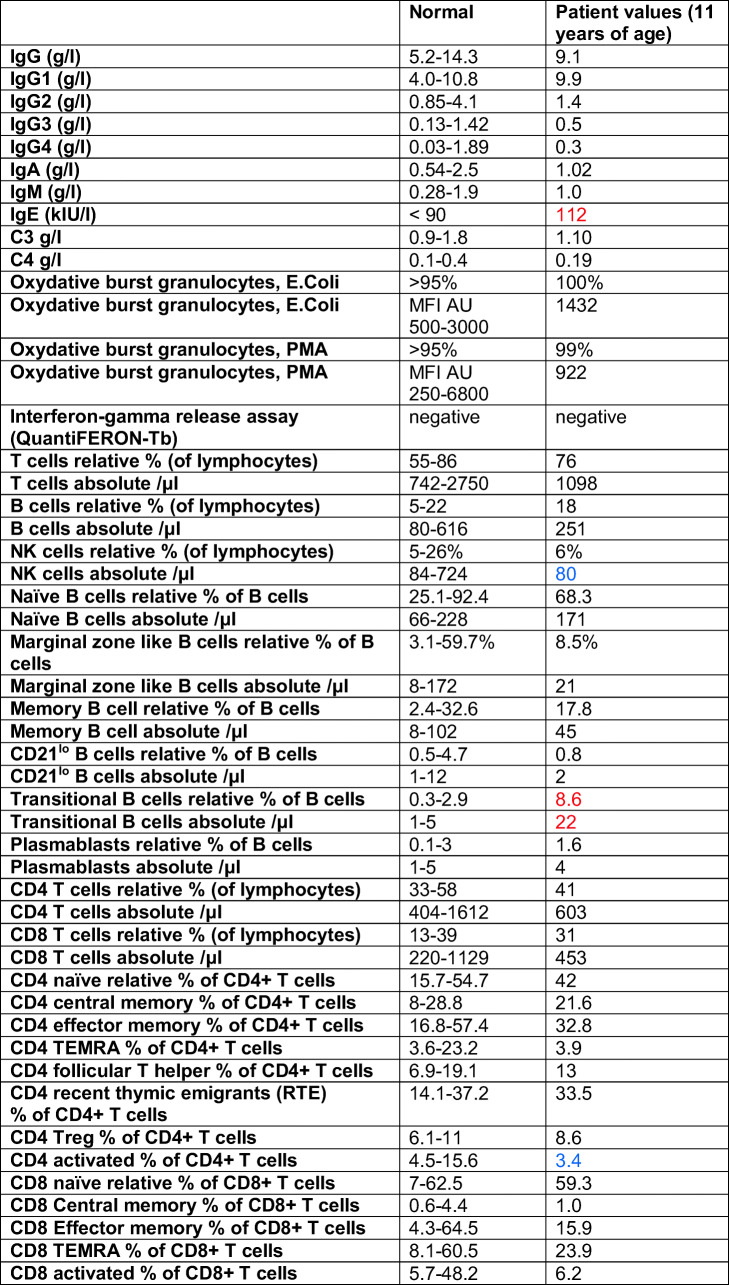
Immunologic evaluation

Immunoglobulins include IgG subclasses show age-specific normal values. Lymphocyte, T and B cell subpopulations, and neutrophil burst assay have normal values validated in healthy adult individuals. Numbers above reference values are indicated in red, numbers below reference values are indicated in blue

Since POLA1 converts immune-stimulatory RNA into more inert RNA/DNA hybrids, XLPDR is associated with increased interferon formation and augmented interferon-stimulated gene (ISG) expression. ISG expression was first measured in peripheral blood of our index patient in August 2019, in the absence of active infections (Fig. 1c). Five of six tested ISG were slightly elevated when compared to several healthy controls but not to the extent of ISG measured in a patient with a genetically proven SAMHD1-related interferonopathy (Fig. 1c).

We tested the patient’s colon biopsies for evidence of intramucosal excess of interferon production and detected upregulated expression of MX1, a prototypic ISG-protein, in epithelial and immune cells (Fig. 1d). Upregulated epithelial MX1 expression was absent in non-inflamed colon but was also detected in the colon of a non-POLA1-associated inflammatory bowel disease patient (Fig. 1d). The latter is in keeping with recent analysis of colon epithelium ISG expression in patients with both active Crohn’s disease and ulcerative colitis [4]. Given that type I interferon receptors act through a Janus kinase (JAK)/signal transducer and activators of transcription (STAT) signaling cascade, treatment of the index patient was started with the JAK1/3 inhibitor tofacitinib as an immune-modulating monotherapy (10 mg daily for 14 days, afterwards 5 mg per day) in December 2019. Abdominal pain decreased and fecal calprotectin, a biomarker of mucosal inflammation, declined from initially 431 μg/g to 96 μg/g (near the normal range) after 8 weeks and remained low at 76.2 μg/g when controlled after 6 months of tofacitinib monotherapy. CRP was below 3 mg/l and blood sedimentation rate was 1 mm/h when measured after 8 months of tofacitinib monotherapy. There was a tendency of reduced ISG in peripheral blood when re-assessed after 8 weeks and 8 months of JAK inhibition (Fig. 1c). No bacterial or opportunistic infections occurred during the first 8 months of tofacitinib treatment. A clinical examination after 8 months of tofacitinib by an ENT specialist demonstrated absence of inflammation endonasal, in the epipharynx and in the middle ear. His current weight is 31.1 kg, and his current height is 137.9 cm (both 5th percentile for the current age). To our knowledge, this is the first XLPDR patient treated with a JAK inhibitor, associated with a clinical response without adverse symptoms or side effects on laboratory parameters. This expands the previously demonstrated potential of these drugs in monogenic interferonopathies [5] to possible use in XLPDR patients with uncontrolled autoinflammatory manifestations.

This is the first genetically confirmed XLPDR patient reported that has bowel histopathology available. Up until now, reports consisted of persistent diarrhea in infancy that was infrequently fully investigated. This case, along with a Spanish patient with jejunal inflammation resembling jejunal Crohn’s disease [6], demonstrate that XLPDR can be accompanied by chronic gastrointestinal inflammation. The patient conformed to the previously described immune phenotype of XLPDR. He did not have antibody deficiency which would have explained the susceptibility to airway infections. Follicular T helper cells and memory B cells were also normal in our patient. It has recently been shown that NK cells are quantitatively and functionally altered in patients with XLPDR [3]. In line with these data, our patient had slight NK cell cytopenia and a relative paucity of mature CD56^dim^ NK cells in peripheral blood. Functionally, NK cell degranulation, as assessed by CD107a expression, was more preserved than IFN-γ formation following receptor-dependent activation. Receptor-independent-NK cell activation was normal, suggesting a defect in the immune synapse formation that depends on the surface receptors [3]. We have not experimentally studied synapse formation which has been demonstrated to be abnormal in NK cells of XLPDR patients. The quantitative and qualitative NK cell abnormalities may contribute to the susceptibility to infections in our patient, although functional in vivo redundancy of innate lymphoid cells has been demonstrated [7].

In summary, XLPDR is a very rare disease. The XLPDR patient reported here contributes to our understanding of the clinical and immunological spectra of the disease and generates preliminary evidence of the efficacy of JAK inhibition.

## Electronic Supplementary Material

Figure S1 Length and weight for age during the first two years of life in the index patient using WHO percentile curves. (PDF 17228 kb)

Figure S2 NK cell phenotype: PBMC derived NK cells were enumerated by staining for CD3 and CD56 and flowcytometric analysis. CD3^−^CD56^+^ NK cells were subdivided into the CD56^hi^ and CD56^dim^ sub-compartments. (PDF 546 kb)
