# Supplementary material for: JAK Inhibition in a Patient with X-Linked Reticulate Pigmentary Disorder
Source: J Clin Immunol. 2020 Sep 28;41(1):212–6. doi: 10.1007/s10875-020-00867-7 (PMC7846528; doi:10.1007/s10875-020-00867-7)
Supplement: Supplementary file 1 — Length and weight for age during the first two years of life in the index patient using WHO percentile curves. (PDF 17228 kb) [file 10875_2020_867_MOESM1_ESM.pdf]

Length-for-age BOYS

Birth to 2 years (percentiles)

World Health Organization

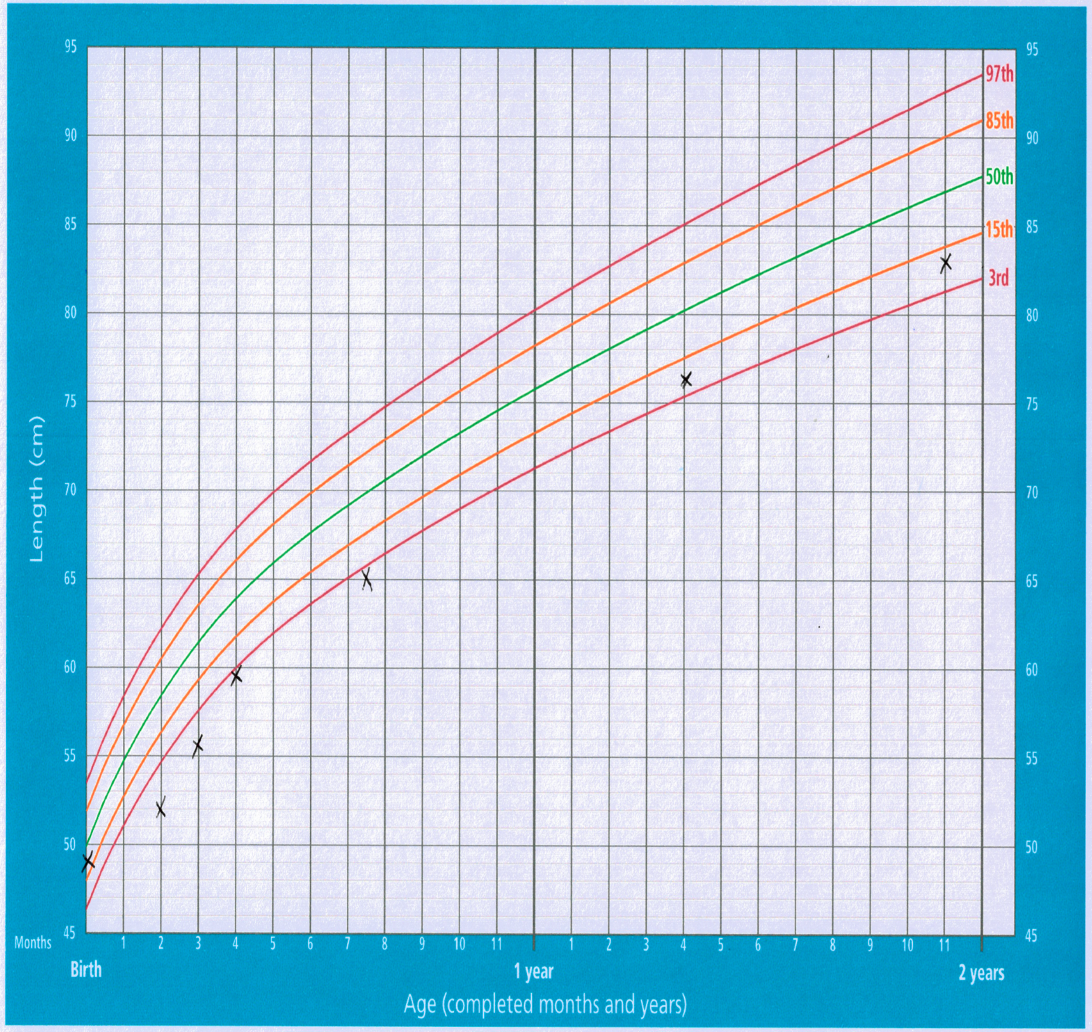

WHO Child Growth Standards

Weight-for-age BOYS

Birth to 2 years (percentiles)

World Health Organization

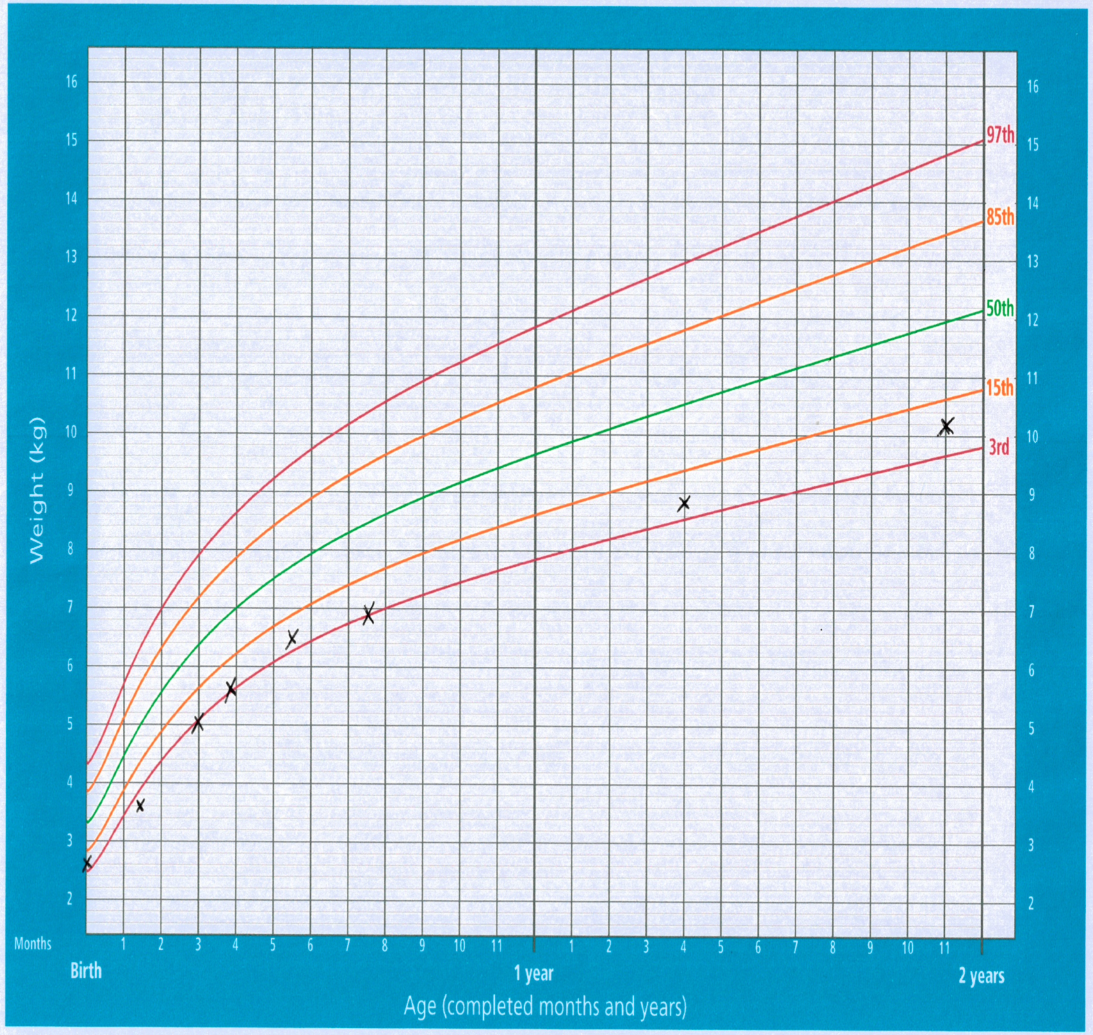

WHO Child Growth Standards
