# Supplementary figures and images for: JAK Inhibition in a Patient with X-Linked Reticulate Pigmentary Disorder
Source: J Clin Immunol. 2020 Sep 28;41(1):212–6. doi: 10.1007/s10875-020-00867-7 (PMC7846528; doi:10.1007/s10875-020-00867-7)

Figure S2

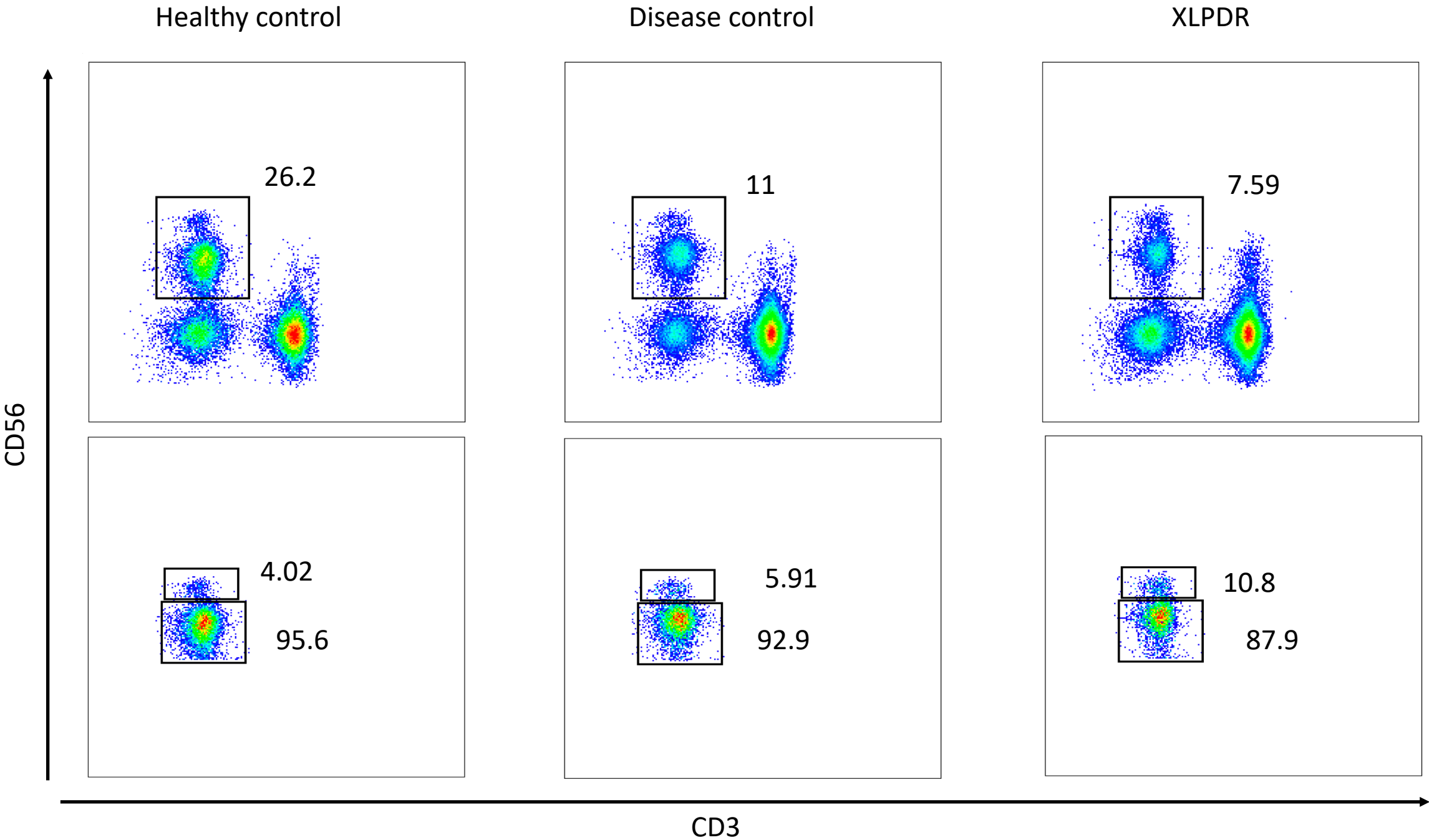

Supplement: Supplementary file 2 — NK cell phenotype: PBMC derived NK cells were enumerated by staining for CD3 and CD56 and flowcytometric analysis. CD3−CD56+ NK cells were subdivided into the CD56hi and CD56dim sub-compartments. (PDF 546 kb) [file 10875_2020_867_MOESM2_ESM.pdf]
